# HARNAS: Human Activity Recognition Based on Automatic Neural Architecture Search Using Evolutionary Algorithms

**DOI:** 10.3390/s21206927

**Published:** 2021-10-19

**Authors:** Xiaojuan Wang, Xinlei Wang, Tianqi Lv, Lei Jin, Mingshu He

**Affiliations:** School of Electronic Engineering, Beijing University of Posts and Telecommunications, Beijing 100876, China; wxl2019@bupt.edu.cn (X.W.); lvtianqi@bupt.edu.cn (T.L.); jinlei@bupt.edu.cn (L.J.); hemingshu@bupt.edu.cn (M.H.)

**Keywords:** deep learning, human activity recognition, multi-objective optimization, multimodal sensor data, neural architecture search

## Abstract

Human activity recognition (HAR) based on wearable sensors is a promising research direction. The resources of handheld terminals and wearable devices limit the performance of recognition and require lightweight architectures. With the development of deep learning, the neural architecture search (NAS) has emerged in an attempt to minimize human intervention. We propose an approach for using NAS to search for models suitable for HAR tasks, namely, HARNAS. The multi-objective search algorithm NSGA-II is used as the search strategy of HARNAS. To make a trade-off between the performance and computation speed of a model, the F1 score and the number of floating-point operations (FLOPs) are selected, resulting in a bi-objective problem. However, the computation speed of a model not only depends on the complexity, but is also related to the memory access cost (MAC). Therefore, we expand the bi-objective search to a tri-objective strategy. We use the Opportunity dataset as the basis for most experiments and also evaluate the portability of the model on the UniMiB-SHAR dataset. The experimental results show that HARNAS designed without manual adjustments can achieve better performance than the best model tweaked by humans. HARNAS obtained an F1 score of 92.16% and parameters of 0.32 MB on the Opportunity dataset.

## 1. Introduction

Human activity recognition (HAR) refers to the process of collecting physical information generated from human movement to identify human activities, and it has been widely used in smart healthcare [1] and other fields. Non-wearable monitoring technology and wearable monitoring technology are the two main ways to obtain data through sensors. In particular, monitoring methods based on low-power wearable devices tend to be more robust and accurate due to their reliance on contact measurements, which offer great potential for improving object recognition and understanding. For analysis methods based on such low-power wearable devices, classification accuracy and high efficiency need to be considered simultaneously.

### 1.1. Research Motivation

Traditional sensor-based HAR systems adopt machine learning (ML) methods. The performance of these methods mainly depends on the feature extraction performance. Among hand-extracted features, there is often a large amount of redundancy; as a result, additional features extracted from the time and frequency domains do not necessarily contribute to improving the recognition accuracy. Therefore, in most previous work, researchers have chosen to reduce the dimensionality of the extracted feature vectors by using techniques such as linear discriminant analysis (LDA). In addition, the ML-based approach does not work well for complex HAR tasks.

Recently, researchers have begun to focus on the potential applications of HAR models based on deep learning (DL) [2], which can automatically extract salient features through different filters for recognition. In [3], salient features were extracted by a deep neural network (DNN) from hierarchical representations. DL technology is playing an important role in advancing the capabilities of activity recognition. Prominent examples of this technology include convolutional neural networks (CNNs) and recurrent neural networks (RNNs). For typical large-scale time-series-based classification tasks such as HAR, CNNs exhibit translation invariance, which is a highly beneficial feature for image classification. RNNs can help to obtain global information for activity recognition, thereby improving the accuracy achieved on complex HAR tasks. The backpropagation through time (BPTT) method is used to optimize RNNs. Differently from the common backpropagation (BP) algorithm, the loss function in an RNN depends on the values in the output layer at the current moment and the next moment, which may lead to the problem of gradient disappearance or gradient explosion.

**Figure 1:**
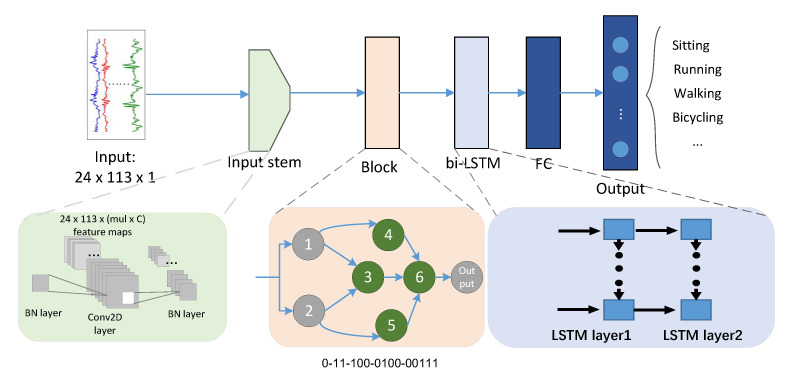
This is an illustration of HARNAS. The top part of the figure shows the overall network structure, and the bottom part shows the structural decomposition of each layer. To reduce the computational complexity, we constrain the search space such that the searchable part of each architecture is the (pink) block, and the rest is fixed. The searchable operation pairs in the block are numbered 3, 4, 5, and 6 (i.e., green), and those labeled 1, 2, and output (i.e., gray) are fixed. Node 6 is called a Concat node. Bi-LSTM is a two-layer LSTM structure, and FC denotes fully connected layers.

The DilatedSRU model [4] adds three dilated CNN layers to extract discriminative features. In order to model the long-term dependence of local features, a new recursive model called the SRU layer is spliced based on CNN. DeepconvLSTM [5] is a deep learning framework composed of a CNN layer and an LSTM layer, which can automatically learn feature representation and model the time dependence between activities. These related works show that a hybrid DL model combining the characteristics of CNNs and LSTM networks can be effectively used to solve complex activity recognition problems. As shown in Figure 1, the pre-model (Input stem), intermediate searched block (Block), and post-model (bi-LSTM) are introduced into the basic model of HARNAS. The pre-model and post-model are the CNN layer and LSTM network, respectively. The CNN layer is used as a feature extractor to extract the abstract representation of sensor data and generate a feature map. After perceiving the local features through the pre-model and the middle search model, the LSTM network synthesizes the local information at a higher level to obtain the global information. However, constructing a strong network model requires a great deal of expert time for manual design because of the very large parameter search space for model construction.

The emergence of automated machine learning (AutoML) [6,7] has yielded automatic methods for handling most of the steps of DL-based model building, including automated data cleaning (autocleaning), automated feature engineering (AutoFE), and neural architecture search (NAS). NAS is a process for automatically constructing a network structure. In most previous work [8,9], NAS has been treated as a single-objective optimization task, in which only the F1 score of the model is considered. In this paper, we not only consider the accuracy of the model, but also adopt the complexity as another important performance metric. Such a multi-objective optimization problem is usually modeled as the process of finding the Pareto optimality. Inspired by the process of biological evolution, a genetic algorithm (GA) is proposed to address this problem.

### 1.2. Main Contributions

The objective of this paper is to make full use of the capabilities of CNNs and LSTM networks to build a fast and accurate HAR model through automatic NAS. The main obstacles to reaching this goal consist of the difficulty of designing an appropriate search space and selecting a search strategy that can converge quickly. When faced with complex and diverse HAR tasks, it is imperative to be able to bypass the process of manual feature engineering and automatically design a network with better performance than can be achieved through manual design to suit the current identification task. In addition, another problem to consider is how to design a lightweight and fast model structure that can enable accurate recognition at the mobile end. For the former purpose, an NAS network based on the non-dominated sorting genetic algorithm (NSGA-II) is adopted. Regarding the latter, in [10], it is considered that element-wise operations are not taken into account in the calculation of the number of floating-point operations (FLOPs); however, although operations of this kind involve a small number of FLOPs, they have a relatively heavy memory access cost (MAC). Therefore, the MAC and the number of FLOPs are both quantities that affect the running speed of a model. Inspired by this, we propose a tri-objective search strategy that considers the weighted F1 score, the MAC, and the number of FLOPs to search for a model with both high precision and high speed.

In summary, our contributions are as follows:

(1) We combine HAR and NAS for the first time and propose HARNAS to realize the liberation of the network architecture search process from manual effort. The customized NAS approach is not actually fully automated, as it relies on a manually designed search space as the source of the search. Therefore, we take an CNN+LSTM infrastructure as the basis for the search space and add dilated convolution and jump join operations for target detection to expand the receptive field without losing intermediate information.

(2) For low-power devices, such as portable smartphones and wearable sensors, both the effectiveness (e.g., accuracy) and efficiency (e.g., model complexity and run time) of a classification method need to be considered. Therefore, we select both a bi-objective set (including the number of FLOPs and the F1 score) and a tri-objective set (additionally including the MAC) as the evaluation objectives for NSGA-II, which is a typical multi-objective evolutionary algorithm (MOEA). Moreover, based on an evaluation of the numbers of parameters of previous models proposed by researchers, we conclude that HARNAS can achieve better identification of the F1 score with fewer parameters on the Opportunity dataset.

(3) On the basis of NSGA-II, in-depth search experiments are carried out for Pareto optimization based on the Opportunity dataset. Then, the chosen Pareto-optimal architecture is transferred to the UniMiB-SHAR dataset and trained from scratch, and a weighted F1 score of 75.16% is obtained. Among other recognition algorithms applied to the UniMiB-SHAR dataset, the highest recognition rate is 77.60%, and the lowest is 70.82%; thus, a comparison of these results indicates that the structure found with HARNAS can be successfully ported to other datasets.

The rest of this paper is organized as follows. The Section 2 summarizes relevant HAR- and NAS-related schemes. The Section 3 introduces our proposed search scheme. The Section 4 describes the experimental details. The Section 5 gives the results of the verification experiment and presents thorough comparisons. Finally, the article is summarized in the Section 6.

## 2. Related Work

In this paper, an HAR-oriented NAS algorithm is studied for the first time. We present an in-depth discussion of NAS and HAR from a historical perspective by considering previous studies.

### 2.1. HAR

In early studies on HAR, most of the work focused on how to extract effective features from the time and frequency domains to identify human activities. Explicit feature extraction for HAR can be realized through traditional ML and DL methods.

ML-based HAR schemes, such as support vector machines (SVMs) [11], naive Bayes (NB) classification [12], and K-nearest neighbors (KNN) classification [13], mainly rely on manually extracted features. The high-dimensional features extracted from the sensor data are sent to the classifier after dimensionality reduction through principal component analysis (PCA) [14]. Therefore, the results of feature selection will strongly affect the recognition performance.

DL-based recognition algorithms can offer improved performance, in part because the feature extraction process no longer relies on manual effort, but rather is automatic. In [15], a CNN was used to recognize human activities, and the middle layer was used as a hierarchical feature extractor to extract features with a clear hierarchy and rich information from the data. However, a CNN’s natural disadvantage is that it cannot extract global time features or mine time-series features. Deep recurrent neural networks (DRNNs) [16] adopt unidirectional, bidirectional, and cascade structures based on the LSTM architecture, which can capture long-range dependencies in input sequences whose lengths are not fixed. Experimental results show that when the input data of interest are sensor data, the performance of DRNNs is better than that of CNNs.

Hybrid HAR schemes can achieve more efficient and accurate recognition. In the HAR field, DeepConvLSTM [5] was proposed by combining the convolution and LSTM approaches for the first time and achieved state-of-the-art performance. Based on previous experience, we adopt a network structure combining the features of CNNs and LSTM networks, where the CNN structure is obtained through NAS and the LSTM structure is fixed.

### 2.2. NAS

DL models have achieved good results on HAR tasks, but the parameter adjustment process is very difficult. It is worth studying how to combine a large number of hyperparameters and network structure parameters to achieve ideal performance. The ability of NAS to automatically generate networks has become a popular new focus of research. In the search space, it is necessary to determine the number of layers, the operation type of each layer, and the corresponding hyperparameters. The evaluation metrics are defined and the hyperparameters are optimized on the basis of different search strategies. Therefore, the following analysis addresses the search space, search strategies, and evaluation strategies.

The primary task for NAS is to define the search space. ENAS [17] includes a controller based on an RNN and a network structure generated by the controller. To design RNN units, ENAS uses a directed acyclic graph (DAG) to represent the NAS search space. Each RNN contains N nodes, and for each node, a choice can be made among four activation functions; thus, there are $N!*4^{N}$ types of DAGs available for searching. DARTS [18] consists of a repetitive set of structures, each consisting of a number of nodes and edges representing operations. These search spaces have a structure similar to that of NASNet. The search space defined by these methods is simple but computationally expensive. However, our task is not complicated, and only one block is needed to achieve good classification performance. Therefore, a simple DAG design is adopted for our search space.

To allow a large search space to be searched effectively, a suitable search strategy can be adopted in NAS to accelerate the search process. This can be realized by means of numerous algorithms, including reinforcement learning (RL) [18,19,20], evolutionary algorithms (EAs) [21,22], and gradient descent [23,24,25]. In the RL-based method, the controller uses an RNN and is rewarded in accordance with the feedback performance of the generated network architecture. When the controller converges, the network model with the best performance is finally output. Based on the function of EAs, the EA-based method searches for architectures in multiple generations. The performance of the models in each generation is used to guide the next generation toward better fitness. Differently from the former two methods, the gradient descent method regards the search space as continuous and uses the BP algorithm to optimize the parameters in the space.

The evaluation of a model can be divided into the evaluation of the model optimization metrics during the search process and the evaluation of the model performance after the search is complete. During the search process, when multiple mutually exclusive objectives are considered, i.e., accuracy, complexity, power consumption, number of arguments, and latency, there is no way that one architecture can outperform all others in all aspects of performance. Therefore, it is necessary to introduce the concept of the Pareto optimality to solve such a problem. Considerable work has been done in previous studies to investigate methods of solving multi-objective problems. NSGA-Net [26] considers two objectives, one for measuring complexity (i.e., the number of FLOPs) and one for measuring model accuracy. SM-NAS [27] balances efficiency with accuracy. The objectives of [28] include accuracy, latency, and energy reduction. We initially select two objectives, namely, model complexity and classification accuracy. Later, the MAC is added as an additional measure to place more emphasis on model efficiency. After the search is complete, the performance of the resulting model and whether it shows the same good performance on other datasets under the same conditions in terms of GPU hardware resources, ambient temperature, etc. can be further evaluated.

## 3. Approach

Resource-constrained mobile devices are usually limited by power consumption, latency, and memory. Therefore, in the actual model design process, not only the model performance, but also the above factors should be taken into account. Usually, when there are conflicts among several objectives, it is impossible for a single solution to fully satisfy all expectations. In this case, a feasible approach is to reasonably measure the target information and select the most promising model in accordance with the particular needs of the application. To design models for the task of analyzing multimodal sensor data and identifying human behavior, we propose HARNAS, a multi-objective architecture search method based on NSGA-II, which is used to automatically generate HAR task-related models. The desired architecture is Pareto optimal in terms of the performance, complexity, and memory consumption of the recognition task. The rest of this section describes in detail the representation used for the searched models and the optimization process of HARNAS.

### 3.1. The Overall Operation of EAs

EAs draw lessons from the evolutionary operation of organisms in nature. They generally include, architecture encoding, population initialization, fitness evaluation, mutation and crossover, and non-dominated sorting. The total combination of an individual’s genes constitutes the genotype of an organism, while the traits that an individual exhibits are called phenotypes. From the perspective of theoretical analysis, the individual genotype is formed in the form of binary coding so as to define the search space size (see Section 3.2 for details). In order to make the initialization operation as simple as possible, decimal coding is used to define the expressiveness of an individual population and complete the initialization process of the population (see Section 3.3.1 for details). The fitness value of an individual of the initial population is calculated according to the optimized objective function (see Section 3.3.2 for details), and an individual of each generation is selected by a non-dominated sorting algorithm according to the fitness value (see Section 3.3.4 for details). The selected individuals are used as parents to generate the next generation of individuals by performing crossover and mutation algorithm operations on the parents (see Section 3.3.3 for details). The new generation of individuals carry out the next round of iterative operations until the number of iterations reaches the set maximum.

### 3.2. Architecture Encoding

From a biological point of view, each architecture can be defined by a coded representation of genotype and phenotype. In an EA, the whole optimization process is conducted by operating on genes (made up of zeros and ones) that theoretically encode the network structure and the size of the search space. The corresponding transformation process is called encoding. As in the case of NSGA-Net [26], each possible model is regarded as a combination of layer-wise computation blocks. However, for our task, only one network layer (i.e., setting the number of blocks to 1) is needed to achieve good recognition performance. Specifically, we let Λ denote the block of interest. We divide Λ into $n_{p}$ operation pairs based on the depth and width of the whole architecture and obtain:

(1) $$\Lambda =(A_{1},A_{2},\ldots ,A_{n_{p}})$$

where Λ is expressed as a DAG representing the block. $A_{i}$ is defined as the operation pair (α${}_{cj}$,α${}_{oj}$) such that the architecture is encoded with discrete integer values. $\alpha_{cj}$ and $\alpha_{oj}$ denote the operation acting on the current node and the current node of information flow, respectively. The relationship between the members of each operation pair can be obtained from the encoding. The encoding rules are as follows: The nodes are sequentially sorted and encoded within the block. For example, node $\alpha_{oj}$ is represented by a (j−1)-bit binary encoding, where each bit represents whether this node is connected to the previous node (values of 0 and 1 represent whether there is a connection relationship). An example of such an encoding is shown in Figure 1. Finally, we refer to the nodes that have been assigned an operation function in the modeling process as normal nodes, and to the nodes that have no operation function as Concat nodes, which are used for the final output. Collectively, they are all referred to as nodes.

For a predetermined operation pair, the search space is defined by genotype as follows:

(2) $$\Omega =n_{p}\times 2^{\left(1+n_{o}\right)n_{o}/2}$$

where $n_{o}$ denotes the number of nodes. Each operation is recoded with values of 0 and 1, so the number of all operations is $n_{p}$. The overall architecture is shown in Figure 1. Except for the block part, the structure of other parts is fixed. The number of blocks and the number of nodes in a block are super parameters that can be searched (see the introduction below Figure 1 for details). The original number of channels (*C*) of input images is a searchable parameter, which is then expanded to mul times the original number of channels in the Input stem. In the field of image segmentation, an empty convolution operation is used to enlarge the receptive field without sacrificing spatial resolution. To take advantage of these special operations in the HAR domain, we redesign the search space accordingly. The detailed operations are shown in Table 1.

**Figure 2:**
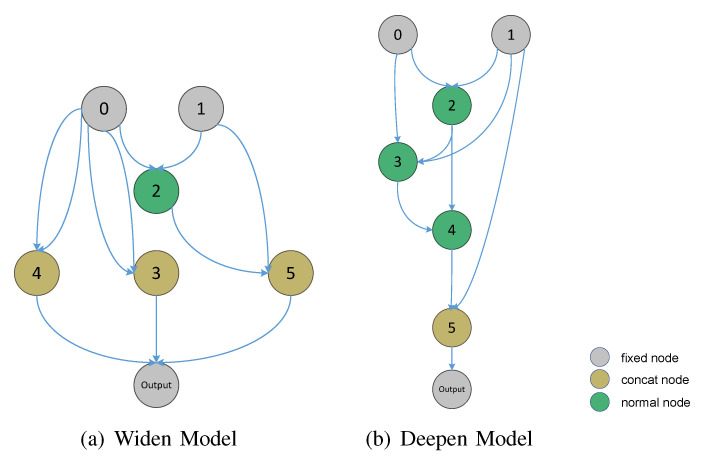
Illustrations of adding nodes to achieve a deeper or wider model structure. (**a**) This is the widen model because there are multiple Concat nodes in the last layer to connect the output nodes. (**b**) This is the deepen model because there is only one Concat node connected to the output node in the last layer, which deepens the number of layers connected to the normal nodes.

The authors of [29] assert that deep structures can realize some functions that cannot be represented in shallow structures. When building a model to solve complex problems, the features can be better fit by increasing the number of structural layers, i.e., deepening the model. At the same time, it is easier for each layer to learn the feature transformation. Can we similarly achieve better model capabilities by increasing the width of the model? The Concat node is the final output node of the model, as shown in Figure 2. If there are N Concat nodes in the same layer, the final result of the model is the concatenate of these N nodes, which increases the width of the model. In addition, the normal and fixed nodes in the model are connected together through Add operation. Accordingly, the fewer Concat nodes there are, the deeper the depth of the model. Therefore, in the following experiments, we use the number of Concat nodes to represent the model depth.

### 3.3. Optimization Procedure

The network structure searched using HARNAS is a generation that is collectively called the population. In the initial generation stage, population networks (PNs) are constructed from randomly generated genotypes, where each individual’s genotype represents a network model. In the evaluation stage, the generated individuals are trained as identifying networks (INs). After training, the individuals are evaluated according to a fitness value (F) function, which is used to measure whether they should continue to participate in the iterative process. According to the evaluation results, the initial individuals are assigned to different levels of the Pareto optimal set by non-dominated sorting, and the first N solutions are obtained by sorting the initial population. The evolution of the offspring is completed by crossing and mutating these N individuals of the parent generation, and the new population is formed by merging the offspring and the parents. This process is repeated until the set termination conditions are met. Algorithm 1 presents the pseudocode of HARNAS. Below, the four major components of HARNAS are further described: the initial population, the fitness function, the crossover and mutation operations, and the non-dominated sorting process.
**Algorithm 1** HARNAS Search Algorithm for Neural Architectures**Input:** Search space (e.g., hyperparameters including layers and blocks), fitness value (F), $n_{population}$, $n_{epochs}$
  **Output:** Best identifying network (IN${}^{*}$) of the Pareto optimal set.
1:Initialize the population networks (PNs);2:**for** each $i\in [1,n_{population}]$ **do**3:  **for** each j∈[1,PNs] **do**4:    Train $IN_{j}$ for $n_{epochs}$;5:  **end for**6:  Update PNs with *F* with the EAs;7:**end for**8:**return**${\left(PN\right)}_{i}$=$n_{generations}$


#### 3.3.1. Initial Population

Despite our analysis of the network structure genotypes in subsection A, we find that genotypes expressed with binary encoding have too many bits and are too complicated for use in practical applications. Therefore, we instead encode the network architectures with a decimal-coded representation based on phenotypes to reduce the number of representation bits. A population of different phenotypes is randomly generated subject to the following restrictions:

(3) $$len\left[f\left(x_{k}\right)\right]=4\times {nodes}_{max}+1.$$

(4) $$0\leq f\left(x_{k}\right)\leq \left\{\begin{array}{cc} \lfloor \frac{n_{1}}{2}\rfloor +1 & k=2n_{1}\\ n_{ops}-1 & k=2n_{2}+1\\ 40+8\Delta V_{x_{k}} & k=len[f\left(x_{k}\right)]-1 \end{array}\right.$$

where $x_{k}$ represents the *k*th bit in the phenotype. Restriction (3) defines the length of a phenotype, where ${nodes}_{max}$ represents the total number of normal and Concat nodes. Restriction (4) defines the range of values for each bit in the coded representation of a phenotype, where $n_{1}$ is limited to $\left(0,1,\ldots ,\frac{len[f\left(x_{k}\right)]-3}{2}\right)$, $n_{2}$ is limited to $\left(0,1,\ldots ,\frac{len[f\left(x_{k}\right)]-3}{2}\right)$, and $\Delta Vx_{k}$ is limited to [0,1,2,3]. $n_{ops}$ represents the total number of operations.

The maximum and minimum bounds of these restrictions can be modified, and the variable parameters (i.e., $\Delta V_{x_{k}}$, ${nodes}_{max}$) can also be modified to adapt to HAR tasks with different data volumes.

#### 3.3.2. Fitness Function

To consider model performance and efficiency at the same time, the following three objective functions are minimized in this study to evaluate the optimal selection of an HAR model.

First Objective Function: The first objective function considers only the model performance. In general, we use accuracy and complexity to judge the training results for a neural network. However, for unbalanced datasets (such as the Opportunity dataset, in which 75 % of the data are in the Null class), even a high accuracy or a low error is not sufficient to confirm the merits of the identification results. Therefore, we choose to calculate the precision and recall based on the confusion matrix and obtain the F1 score as a comprehensive metric. This method can distinguish the model performance on unbalanced data and yield a fair score. The precision and recall are calculated as shown in the following formulas:

(5) $$Precision=\frac{TP}{TP+FP}$$

(6) $$Recall=\frac{TP}{TP+FN}$$

where TP, FP, and TN represent the numbers of true positives, false positives, and false negatives, respectively. The precision represents the proportion of positive samples correctly predicted by the model, while the recall represents the proportion of all actually positive samples identified in the predicted results. To accurately predict the identification accuracy on an unbalanced dataset, the balanced F1 score, which is a weighted average of the precision and recall, is adopted. Its definition is as follows:

(7) $$F1\left(c_{i}\right)=\frac{Precision_{c_{i}}*Recall_{c_{i}}}{Precision_{c_{i}}+Recall_{c_{i}}}$$

(8) $$\;F1(x)=\sum_{i=1}^{M}\left(F1\left(c_{i}\right)*\omega_{i}\right)$$

In this way, we obtain the first objective function of the HARNAS model, as given in Equation (8). Here, $c_{i}$ represents the *i*th class, *M* represents the total number of classes, and $\omega_{i}=\frac{N_{i}}{N}$ represents the proportion of samples labeled as belonging to the *i*th class.

Second Objective Function: There are many factors that can affect the complexity of a model to varying degrees, such as the depth of the model, the width of the model, and the number of channels per layer. To comprehensively evaluate the model complexity, we choose the number of FLOPs as a proxy for network complexity.

Evaluating the FLOPs-based cost mainly involves considering the convolution cost ($C_{conv}$), the full connection cost ($C_{full}$), the activation function cost ($C_{a}$), and the LSTM cost ($C_{lstm}$), as follows:

(9) $$F_{2}\left(x\right)=C_{conv}+C_{full}+C_{a}+C_{lstm}.$$

To calculate the number of FLOPs for a convolution operation, we write the following:

(10) $$C_{conv}=2K_{h}\times K_{w}\times C_{in}\times C_{out}\times H\times W$$

where $C_{in}$ represents the number of input channels, and $K_{h}\times K_{w}$ represents the size of the convolution kernel. $C_{out}$ represents the number of output channels, and H×W represents the size of the feature maps.

Since the weights are not shared in the full connection operation, the number of FLOPs for a fully connected layer is calculated only as a function of the numbers of input and output nodes:

(11) $$C_{full}=2N_{in}\times N_{out}$$

where $N_{in}$ and $N_{out}$ represent the numbers of input and output nodes, respectively.

Activation functions may contain a variety of combinations of algebraic operations, so the numbers of FLOPs for different activation functions are different. Table 2 lists the FLOP counts for common activation functions.

For the calculation of FLOPs for an LSTM layer, we combine it with an LSTM cell [30] to obtain the following:

(12) $$\begin{array}{cc} \; & C_{lstm}=3\left(4f_{b}\left(x\right)\right)+5f_{b}\left(x\right)+9f_{c}\left(x\right),\\ \; & f_{b}\left(x\right)=\left(W_{x}\left[2\right]+W_{h}\right)\times W_{h}\\ \; & f_{c}\left(x\right)=W_{x}\left[0\right]\times W_{x}\left[1\right]\times W_{h} \end{array}$$

Four nonlinear transformations are included in an LSTM cell to control the state and output at different times. Three of the four nonlinear functions are sigmoid activation functions, and the other one is a tanh activation function. Firstly, the 3D input vector is concatenated with the hidden state vector of the previous moment, and then the concatenated vector is multiplied by the weight matrix. The product result is then passed through sigmoid or tanh function. $f_{b}\left(x\right)$ represents the basic unit of operations for concatenation and multiplication, where $W_{h}$ is the dimensionality of the hidden layer and $W_{x}$ is a three-dimensional array in which the different dimensions correspond to the length of the input vector, the number of batches, and the number of features per input vector. According to Table 2, for the output results after the sigmoid activation function, the FLOP count obtained is $4f_{b}\left(x\right)$. Similarly, the FLOP count for a tanh activation function in an LSTM cell is $5f_{b}\left(x\right)$.

In addition to the four activation functions in the gate structure, an LSTM cell includes three matrix multiplications, an addition operation, and a tanh activation function. The size of the feature map after the gate function calculation is the same, and the result is as shown for $f_{c}\left(x\right)$.

Third Objective Function: To reduce the number of parameters in the model and shorten the run time, the number of FLOPs is adopted as an evaluation metric. However, it was pointed out in [10] that the number of FLOPs, which is an indirect metric of the computing speed, cannot completely reflect the run time. The MAC is also a factor affecting model speed; therefore, we add the MAC as a third search metric.

To calculate the MAC of each layer in a network, three components must be considered: (1) $MAC_{input}$: the cost of reading the input vector from the main memory (or reading the feature map if not the first layer); (2) $MAC_{weight}$: the cost of reading the weights from the main memory and computing the dot product; (3) $MAC_{output}$: the cost of writing the output vector (or writing the feature map if not the last layer) back to the main memory. Thus, the third objective function is defined as follows:

(13) $$F_{3}\left(x\right)=MAC_{input}+MAC_{weight}+MAC_{output}$$

Ordinary convolution operations, dilated convolution operations, and pooling operations with different convolution kernel sizes are all defined in the search space. The MACs corresponding to these operations are calculated below. For the operation of an ordinary convolutional layer, the computational components of the MAC are as follows:

(14) $$\begin{array}{cc} \; & MAC_{conv\_input}=H_{in}\times W_{in}\times K_{h}\times K_{w}\times C_{in}\times C_{out},\\ \; & MAC_{conv\_weight}=K_{h}\times K_{w}\times C_{in}\times C_{out}+C_{out},\\ \; & MAC_{conv\_output}=H_{out}\times W_{out}\times C_{out},\\ \; & where\;H_{out}=\frac{H_{in}-K_{h}+2P}{S}+1,\\ \; & W_{out}=\frac{W_{in}-K_{w}+2P}{S}+1 \end{array}$$

The meanings of $C_{in}$, $C_{out}$, $K_{h}$, and $K_{w}$ are the same as in Equation (10). $H_{in}\times W_{in}$ and $H_{out}\times W_{out}$ represent the sizes of the input and output feature maps, respectively. *P* and *S* are the padding and stride values, respectively.

Dilated convolution means injecting holes into the standard convolution map to increase the reception field. The method of calculating the MAC is the same as in Equation (14), but the size of the convolution kernel is affected by the dilation rate *D* as follows:

(15) $$K_{dil}=\left(D-1\right)\left(K+1\right)+K$$

where *K* is the size of the original convolution kernel. General convolution corresponds to the special case of D=1.

There are no weight parameters for a pooling layer, so only two components, $MAC_{input}$ and $MAC_{output}$, need to be calculated when calculating the MAC of a pooling layer, and the size of the feature map is the same as in the calculation of $W_{out}$ and $H_{out}$ in Equation (14).

#### 3.3.3. Mutation and Crossover

The crossover operator is designed to exchange information between members of the population and thus preserve good genes. After the parents are selected, the genotypes of the offspring are reconstituted through inheritance or variation. The explicit expression of the genotype is the block of the individual. After one bit of the common binary genotype of the parent is inherited, hybridization is achieved by swapping some bits of the parent.

The mutation operator is designed to keep the population diverse. Based on the coding properties, we use bit flipping to change the genotype. Notably, the flipping of one bit can cause the structure of the space to change completely. Therefore, each mutation changes only one bit of the binary sequence, which is sufficient to construct a new architecture.

#### 3.3.4. Non-Dominated Sorting

In theory, to solve a multi-objective problem, a set of optimal solutions is usually needed. Within this set of optimal solutions, no single solution is completely superior to the other solutions in all dimensions (i.e., the defined objectives). Therefore, for a set of solutions that are not dominated by others, dubbed non-dominated solutions, the process of non-dominated sorting generally consists of three steps: (1) Let the set of all members in the current population be *P*, set $S_{1}=\emptyset$, and add each non-dominated solution *p* to $S_{1}$ to form the first Pareto optimal set. (2) Set $P_{res}=P-S_{1}$, and continue to find the second-order Pareto set by finding the non-dominated solution set from $P_{res}$, denoted by $S_{2}$. (3) Repeat the above steps until P=∅.

## 4. Experiment

In this section, we first introduce two benchmark datasets. Then, to overcome the imbalanced distribution of the datasets, two objectives (the F1 score and FLOPs) are used as metrics to evaluate the model performance. Given these multiple criteria, a model with high accuracy and low complexity is obtained by looking for the Pareto optimal set. In addition, to further pursue a more lightweight model, we consider a tri-objective strategy by adding the MAC as a search objective. Finally, we describe the details of the training process.

### 4.1. Benchmark Datasets

Humans interact with the outside world through a variety of physical activities, including running, jumping, stretching, and so on. Therefore, a benchmark dataset for HAR should include as many human actions as possible. Previous researchers have collected datasets of various actions, including the Opportunity [31] and UniMiB-SHAR [32] datasets. The activity recognition datasets we selected all consist of sensor data. This section describes experiments conducted on the two selected sensor datasets.

The Opportunity dataset was collected by placing wearable devices on the upper body, buttocks, and legs of 4 subjects, who were then asked to perform 17 daily activities, including opening and closing drawers and cleaning tables, in a kitchen scenario. Each subject performed six rounds of actions. The first five records are called activities of daily living (ADLs), and the last record required the subject to perform a scripted action flow, known as a Drill session. We trained models on the data from all ADL and Drill sessions for the first and fourth subjects, as well as the ADL1, ADL2, and Drill sessions for subjects 2 and 3. We finally evaluated the classification performance on the ADL4 and ADL5 test sets for subjects 2 and 3. The ADL3 datasets for subjects 2 and 3 were reserved for validation. Because data can be lost during wireless transmission, we also used linear interpolation to reduce the impact of the missing data in the dataset. The sensor data were acquired from 5 inertial measurement units, 2 InertiaCube3 sensors, and 12 Bluetooth 3-axis acceleration sensors at a frequency of 30 Hz. Each sensor axis was treated as an individual channel, yielding an input space with a dimension of 113 channels.

The UniMiB-SHAR dataset was collected by recording 17 activities performed by 30 volunteers using acceleration sensors in mobile phones. The window size used to obtain the sensor data was 3 s (with a sampling frequency of 50 Hz), and the different actions were divided by the peak values. On the basis of previous studies, 10-fold cross-validation was adopted. This means that all the data were randomly divided into 10 folds, and each fold was taken as the test set in turn, with the remaining 9 folds as the training set. The test results from all 10 rounds were recorded and averaged. In the experiment, we established 10 random seeds.

### 4.2. Model Training

We used the NVIDIA 1080 Ti GPU training model in PyTorch and used the random gradient descent algorithm and the cross-entropy function to train the model for each network generated during the architecture search. Our initial learning rate was 0.025, each model was trained for 25 epochs, and the batch size was set to 128. Then we got the F1 score of the model on the validation set. The optimal model was trained on the test set to obtain the F1 score. The precision of data representation in the network has an influence on the performance. The weight precision of the model used in this experiment was FP32. In the follow-up experiments, the performance difference between FP64 and FP32 format was proved.

### 4.3. Analysis with Peer Competitors

The effectiveness of the model searched by NASHAR is illustrated by comparison with other peer competitors. Five comparison models are proposed. In addition to some conventional models, such as 1D convolution and LSTM, we will focus on the overall ideas of the DeepConvLSTM, dilatedSRU, Hybrid, and HARNAS-searched models.

## 5. Results

### 5.1. Multi-Model Classification

We first compared the performance of HARNAS with the performance of other HAR classification models on the Opportunity dataset. The weighted F1 score was used as the evaluation parameter. Next, the selected optimal model was transferred to the UniMiB-SHAR dataset for training from scratch. The other recognition models considered for comparison are described below.

#### 5.1.1. Convolutional Neural Network (CNN) with 1D-Convolution Kernels

The basic unit of this model [33] consists of a 1D convolutional layer combined with a ReLU activation layer and a maximum pooling operation. This convolution–activation–pooling combination is repeatedly stacked to deepen the network. Finally, a fully connected layer is used to fuse the channel information, and a softmax layer is used to output the probability of each class.

#### 5.1.2. LSTM

This model [33] consists of two LSTM layers. The final classification results are output after a dense layer and a softmax layer. Activation functions are used for the input and memory in the cells.

#### 5.1.3. A Hybrid Model and DeepConvLSTM

The basic idea in [5,33] is the same, namely, to use a hybrid model combining the characteristics of a CNN and LSTM to address the HAR identification task. The main differences lie in the numbers of convolutional layers and LSTM layers.

#### 5.1.4. DilatedSRU

This is a new network [4] proposed to solve the HAR identification problem. Dilated convolution is used to extract local or short-term features, and a recurrent network is used to simulate long-term dependence.

**Figure 3:**
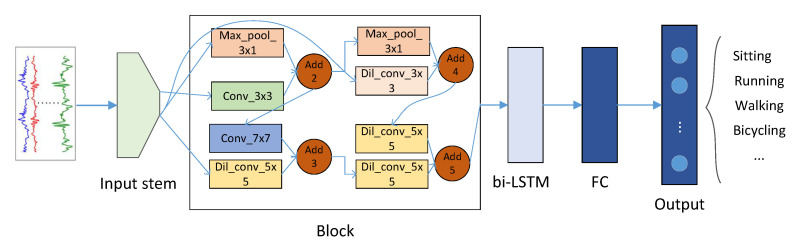
The lightness–accuracy tradeoff based on the HARNAS-searched model.

**Figure 4:**
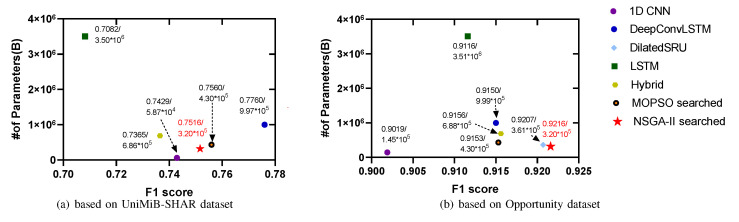
The weight–F1 score tradeoff for different DL models on two benchmark datasets. (**a**) The optimal models searched on the Opportunity dataset are directly transferred to the UniMiB-SHAR dataset for training. (**b**) The model is trained and tested on the Opportunity dataset, and the F1 score and parameter quantity of the model are obtained.

We compared the weight of each model in terms of the number of network parameters. We first searched for the ideal model (as shown in Figure 3) using the NSGA-II method on the Opportunity dataset and then migrated the identified model to the UniMiB-SHAR dataset to test its portability. Figure 4 shows the weight–F1 score tradeoffs for the different DL models on the two benchmark datasets. The hyperparameters we set here included a sliding window length = 1 s and a number of nodes = 4. The setting of the values for these hyperparameters is discussed in subsection D. The F1 scores of the Pareto optimal set found based on HARNAS were the highest, reaching 92.16% on the Opportunity dataset and 75.16% on the UniMiB-SHAR dataset, thus demonstrating that HARNAS can effectively improve both the F1 score and portability of the models found through the search process.

In addition, we replaced the NSGA-II algorithm with the multi-objective particle swarm optimization (MOPSO) algorithm to obtain another set of experimental results, and a comparison with these results further proved that the recognition effect of HARNAS was better. In Figure 4, it can be clearly seen that the models based on NSGA-II exhibited the best tradeoff effect. In Figure 4a, the F1 score of the migrated model is not the highest. However, compared with the DeepConvLSTM model, the number of parameters is two-thirds less, thus achieving a tradeoff between the F1 score and lightness.

### 5.2. Multi-Objective Search

**Figure 5:**
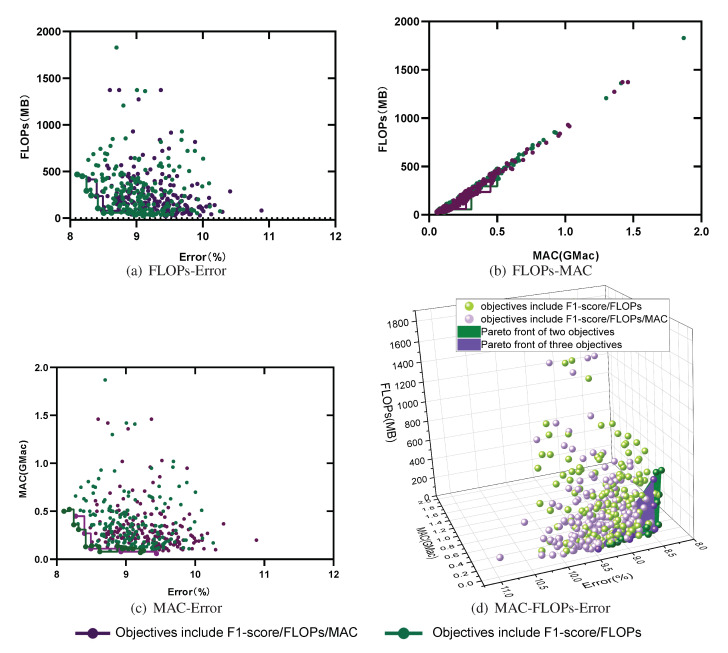
The evaluation results for 200 architectures (20 in each generation, 10 generations in total) found using the NSGA-II method with two objectives (FLOPs and Error) and three objectives (FLOPs, Error, and MAC) on the Opportunity dataset.

To consider both model accuracy and running speed, the model search was extended to two objectives (the bi-objective strategy) and three objectives (the tri-objective strategy). The two metrics considered in the bi-objective case were the number of FLOPs and the F1 score, while in the tri-objective case, the MAC was also included. Figure 5 compares the overall search results and the Pareto optimal fronts (purple and green curves in the lower left corner) in the search space for these two multi-objective tasks based on NSGA-II. The Pareto set of the bi-objective search consisted of 16 architectures, but there were many outliers. The Pareto set of the tri-objective search was composed of seven architectures, and the population was relatively concentrated. In addition, the Pareto optimal front analysis in Figure 5a shows that the frontier of the tri-objective search was closer to the lower right than the frontier of the bi-objective search, that is, the former had both lower complexity and lower accuracy than the latter; analyzing (b) and (c) in the same way reveals that the models found through the tri-objective search were lower in FLOPs and MAC and higher in error. This conclusion can be proven more clearly in Figure 5d.

**Figure 6:**
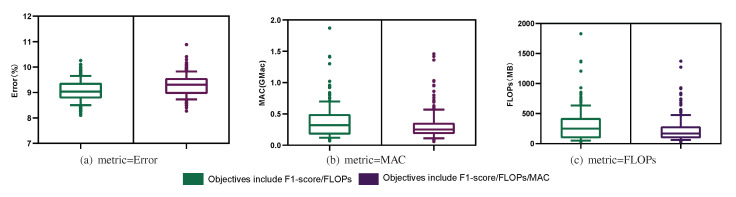
The differences between the bi-objective and tri-objective results for 200 architectures are analyzed in terms of the FLOPs, MAC, and Error metrics.

The model structures on the frontier were the elite models, which reflected the best structures offered by the current population. Therefore, after comparing the frontiers based on different objectives, we performed a box diagram analysis for all model structures in terms of the three metrics. According to the analysis in Figure 6, the 200 models found based on all three objectives were lower in FLOPs and MAC than those found in the bi-objective search, but their error was also higher. This is consistent with the results obtained in Figure 5. The reason for this is that the tri-objective search not only imposed complexity requirements on the sought models, but also limited their memory access speed. These two objectives conflicted with that of reducing the model classification error, thus reducing the probability of finding a model with a low classification error. For example, when a model with a small Error and a low FLOP count but a large MAC was found, it could be considered Pareto optimal in the bi-objective search task, whereas it would be deemed unsatisfactory in the tri-objective search task. Note that in the training process, the weighted F1 score was used as the optimization metric, but when solving for the Pareto optimal set and in the subsequent analysis of the experimental results, we use error=100−(F1score) instead.

### 5.3. How Can Depth, Width, and Model Performance Be Quantitatively Evaluated?

More complex problems can be modeled by either increasing the number of network layers or by building shallower but more compact structures. On the Opportunity dataset, we selected two sets of search objectives, corresponding to bi-objective and tri-objective searches, and designed eight groups of experiments by defining the number of nodes in each block. The evaluation metrics for each group of experiments were defined as the Error, FLOP, and MAC metrics. The different numbers of Concat nodes considered were 1, 2, 3, and 4.

Each set of results shown in Table 3 was obtained by calculating the average value of each of these three metrics for the 200 structures found for each number of Concat nodes. In these eight groups of data, with the deepening of the network (i.e., the reduction of the number of Concat nodes), the network identification performance improved to varying degrees, and the network complexity was also reduced. However, the model memory access speed showed non-monotonic results in some sets of data. For example, in the bi-objective search with nodes = 5, the MAC started to decrease when the number of Concat nodes was 4. From these experiments, it can be concluded that increasing the model depth can indeed improve the model performance to a certain extent. From the MAC perspective, however, deeper is not necessarily better.

**Figure 7:**
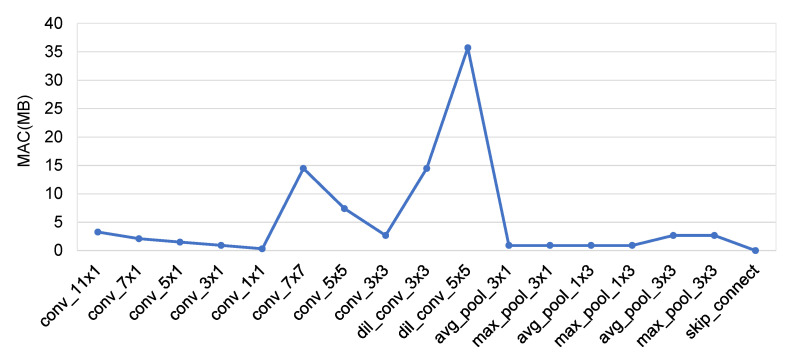
The calculated proxy MACs for 17 operations.

To clarify the uncertain trend of the MAC with an increasing number of nodes, we designed another group of experiments. We calculated the MACs for various operations using Equations (13)–(15). We assumed the hyperparameters needed in the formulas to have the values shown in Table 4 and calculated corresponding proxy MACs for the 17 operations defined in the search space. The proxy MAC of an operation does not represent the true MAC value of that operation in all cases, but it can be used to evaluate the relative contributions of various operations to the overall MAC. Figure 7 shows the proxy MACs of the different operations. It can be seen that when there are operations such as conv_7 × 7, dil_conv_3 × 3, and dil_conv_5 × 5 in a network model, their contributions to the MAC will be relatively large. Therefore, we counted the proportions of the different operations in all network structures with different numbers of Concat nodes to analyze why the MAC would change irregularly with increasing network depth.

**Figure 8:**
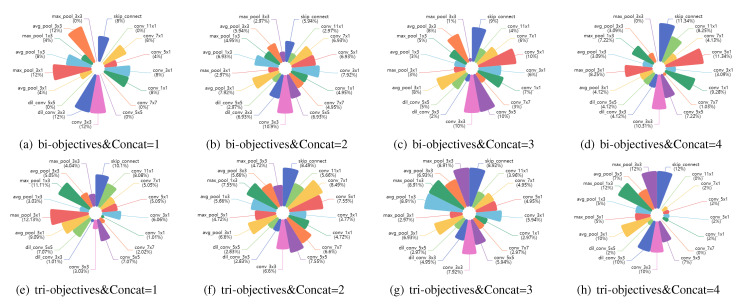
The relative proportions of 17 types of operations in 200 models with five nodes and 1, 2, 3, or 4 Concat nodes found under different search objectives, presented as pie charts.

Since the analysis method is similar, we select only the case in which the number of nodes is 5, meaning that the number of searchable Concat nodes is the largest, to illustrate the analysis of the bi-objective and tri-objective search results here. Figure 8a–d presents the proportions of different operation types in models with 1 to 4 Concat nodes found under bi-objective search conditions. Figure 8e–h shows the same results for the tri-objective search case. As shown in Figure 8a–d, the relative proportion of the operation with the largest MAC (i.e., dil conv 5 × 5) increases from Concat = 1 to Concat = 3 and suddenly decreases when Concat = 4. Similarly, the relative proportion of conv 7 × 7 is reduced to 3% for Concat = 3 and to 1% for Concat = 4. There are also other relatively high-MAC operations that appear in the Concat = 3 case, but not when Concat = 4, which is one of the reasons for the sudden decrease in the MAC. Meanwhile, it is statistically found that the more Concat nodes there are, the fewer network structures can be searched. The most obvious change is that the number of models searched drops from 27.5% to 1.5% with the increase from Concat = 3 to Concat = 4. Therefore, it seems that the number of network architectures searched when Concat = 4 is too small, and there is a deviation in the statistical MAC proportion, which is not universal, resulting in the sudden decrease in the MAC. It can be concluded that since the number of networks searched varies with the number of Concat nodes, the number of high-MAC operations in the network architectures found in the search simultaneously decreases. The MAC will exhibit a sudden decrease as the number of Concat nodes increases.

### 5.4. Hyperparameter Evaluation

To further explore the influence of different parameters on the multi-objective search results, we selected three main hyperparameters, namely, the length of the sliding window, the number of nodes, and the number of original channels. The experiment was carried out on the Opportunity dataset and in both the bi-objective and tri-objective search cases.

#### 5.4.1. Length of the Sliding Window

To ensure fairness, we used the same sliding window sizes of 0.4, 0.8, 1, and 1.5 s in both bi-objective and tri-objective experiments. For sensor data recognition, the selection of the sliding window size in the preprocessing stage is an important problem. If the sliding window is too large, this will result in too much redundant information and will increase the difficulty of recognition. In contrast, if the window size is too small, there may be too few data within a fixed period of time, causing the amount of feature information extracted to be insufficient for accurate recognition. Therefore, in this section, we wish to reveal the influence of the sliding window size on the performance of the models found in the search process through a number of comparative experiments.

**Figure 9:**
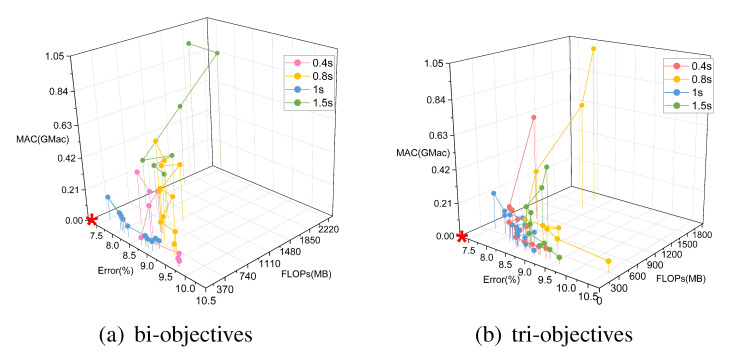
The tradeoff frontiers found with different numbers of optimization targets. (**a**) Pareto optimal frontier under four sliding window sizes in the bi-objective task. (**b**) Based on the tri-objective task.

Figure 9 shows the Pareto optimal sets for the classification behavior on the Opportunity dataset based on the four sliding window sizes in the bi-objective and tri-objective search cases. In Figure 9a, for sliding window sizes of 0.4, 0.8, 1, and 1.5 s, the numbers of Pareto optimal model structures are 8, 11, 10, and 7, respectively. In Figure 9b, the corresponding numbers of Pareto optimal model structures are 13, 9, 17, and 11. When the sliding window size is 1 s, the Pareto optimal set is in the best position, that is, closest to the origin, in both figure panels.

**Figure 10:**
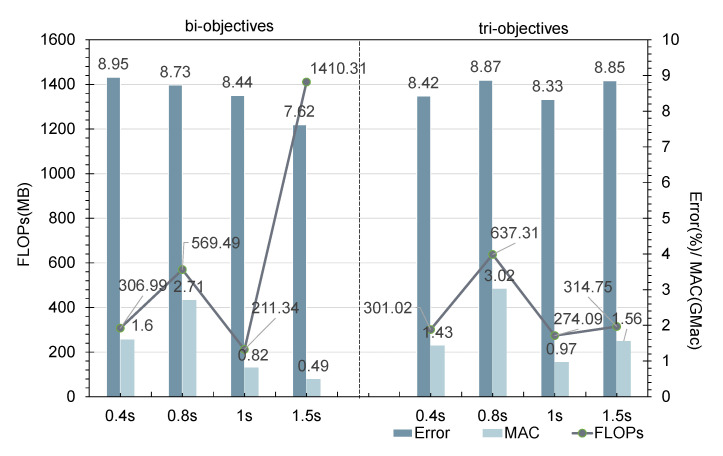
This figure presents a set of comparative experiments. The NSGA-II search strategy was adopted to search model populations with sliding window lengths of 0.4, 0.8, 1, and 1.5 s under different numbers of optimization objectives. The values in the figure were obtained by averaging the results for the structures in the Pareto optimal set in terms of the three objectives.

Figure 10 shows the average values for the Pareto optimal sets found with the NSGA-II-based optimization method under the bi-objective and tri-objective searches with sliding windows of different lengths. For the bi-objective search with a sliding window length of 1 s, the average values of the FLOPs, MAC, and Error metrics are 211.34 MB, 0.082 GMac, and 8.44%, respectively. Compared with the other experiments under the same number of objectives, the classification performance is the best with this sliding window length, and the model complexity and memory-related running speed are the lowest. Similarly, in the tri-objective case, the FLOPs, MAC, and Error metrics are also all minimized when the length of the sliding window is 1 s. Therefore, the performance and efficiency of the architectures in the Pareto set obtained when the sliding window length is 1 s are generally superior.

#### 5.4.2. Number of Nodes

In Section 3, we mentioned that each block consists of several operation pairs, where each operation pair consists of a node and an operation. In this section, we explore the influence of the number of operation pairs on the results of the model search. The number of operation pairs per block directly determines the model complexity. As the number of operation pairs increases, the fitting ability of the model is enhanced, thereby reducing the classification error, but this may also lead to overfitting. If the number of operation pairs is too small, the running speed of the model can be improved, but in extreme cases, this may lead to poor model classification performance. Based on the results of the previous hyperparameter experiment, we set the length of the sliding window to 1 s to carry out this experiment.

**Figure 11:**
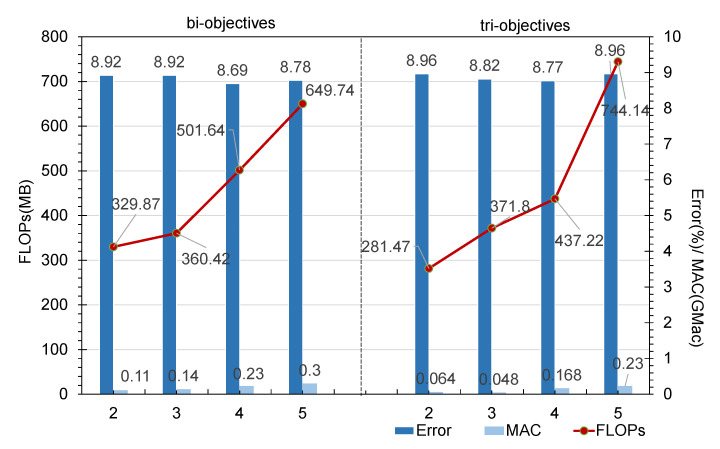
In this group of comparative experiments, the influence of different numbers of nodes in each block on the model search results was tested. The FLOPs, MAC, and Error values were obtained by taking the average over the descendant architectures forming the Pareto optimal set found in the search process.

As shown in Figure 11, for the number of nodes, we selected values of 2, 3, 4, and 5. In both the bi-objective and tri-objective experiments, it was found that the FLOP and MAC metrics always increase as the number of nodes increases. By contrast, the Error metric decreases until the number of nodes reaches 4, and then starts to increase again. Although the FLOP and MAC values continue to grow, it is undeniable that within the acceptable range of FLOP and MAC values, the identification accuracy of the model is a high-priority factor. Therefore, we believe that the best situation is when the number of nodes is equal to 4.

#### 5.4.3. Number of Searched Channels

In the network architecture search phase, the number of original channels was also set as a searchable parameter. The number of original channels is directly related to the number of channels input into the fixed nodes. If the number of channels is too small, each layer can capture only limited features; consequently, even if the network model is deep enough, it cannot extract sufficient information to pass down. However, with an increase in the number of channels, the calculations performed in the model will also increase rapidly, so the ability of a shallow model to extract the features is also very important. To a certain extent, this ability also depends on the number of channels per layer. Therefore, in this section, we study the impact of the number of original channels on the network performance through comparative experiments. The selected search objective set is the tri-objective set. The number of original channels that can be searched is limited to four discrete values: 40, 48, 56, and 64. In addition, based on the results of the above two hyperparameter experiments, we set the length of the sliding window to 1 s and the number of nodes to 4.

As mentioned above, the search space includes the parameter representing the number of channels, and in principle, we should need only to separate 200 individuals according to the number of channels for a particular set of experimental results and then count the results. However, we found that the larger the number of channels is, the smaller the corresponding number of individuals. It would be unfair to measure the Pareto optimal sets for these groups with different numbers of individuals; therefore, we chose a box chart analysis instead. Moreover, in the first two hyperparameter experiments, we found that the variation trends of the FLOP, MAC, and Error metrics were almost the same in the bi-objective and tri-objective cases. Therefore, to avoid repeated experiments, we considered only the tri-objective search case here.

**Figure 12:**
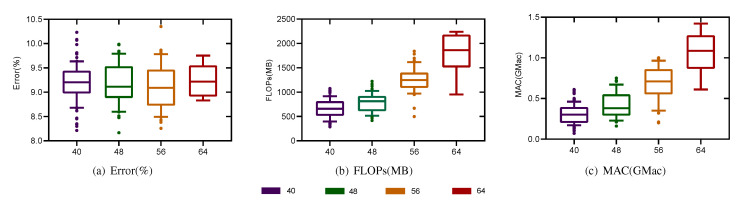
The population found on the Opportunity dataset using the tri-objective search strategy was divided into four groups according to the number of shallow channels. This figure presents the corresponding box charts of the Error, FLOP, and MAC metrics.

As shown in Figure 12, the medians, upper quartiles, and lower quartiles of the Error, FLOP, and MAC metrics were calculated individually according to the number of channels in the 200 architectures found in the search process, and the corresponding box charts were drawn. The results in Figure 12b,c exhibit more obvious differences among different evaluation metrics for the Pareto optimal set of the population found in the search process when divided according to the number of shallow channels. As seen from this figure, as the number of shallow channels increases, the Error metric monotonically decreases, while the MAC and FLOPs metrics monotonically increase. Compared with the case of 48 shallow channels, when the number of shallow channels is 56, the FLOP metric is increased by a factor of 1.8, and the MAC is increased by a factor of 2.2, but the Error shows only a small decrease, corresponding to a large sacrifice in efficiency for a minimal improvement in performance. Therefore, to balance the relationship between performance and efficiency, we believe that the most appropriate number of shallow channels is 48.

**Figure 13:**
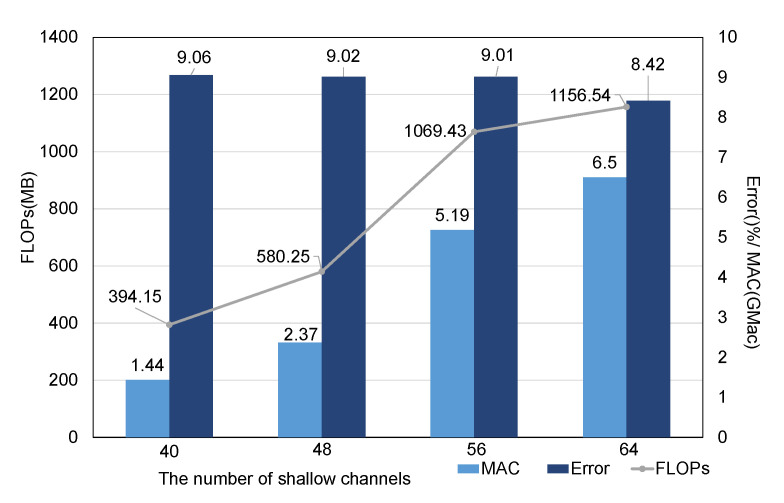
The average evaluation metric values for the Pareto optimal set divided into four groups according to the number of shallow channels.

Figure 13 shows the results of calculating the average values of the experiments. When the number of channels in the shallow network is considered as a searchable parameter in the search space, we find that there is no obvious trend in network performance; that is, more input channels do not necessarily lead to better network performance. However, as the number of channels increases, the network efficiency does show an obvious increasing trend. Accordingly, when we choose fixed network input channel parameters, we should prioritize network models with fewer channels. We also use the number of Concat nodes in each block of the model as a proxy to study the impact of the model depth. The results show that when the number of nodes is 4 and the tri-objective search strategy is adopted, the deeper the model is, the better the model performance and the higher the computational efficiency.

### 5.5. Data Representation Precision

At present, the precision defined by the IEEE 754 standard includes half precision (FP16), single precision (FP32), and double precision (FP64). Single-precision floating-point numbers use four bytes (32-bit binary) to represent a number, double-precision floating-point numbers use eight bytes (64 bits) to represent a number, and half-precision floating-point numbers are 16 bits. Due to the different expressions of floating-point numbers with different precisions, the calculation error is also different. For scientific calculations that need to deal with a large range of numbers and accurate calculation, double-precision floating-point numbers are required. For ordinary multimedia and graphics processing calculations, 32-bit single-precision floating-point calculations are enough. For some applications with lower precision, such as machine learning, half-precision floating-point numbers of 16 bits/8 bits are enough. The experimental results with the FP32 and FP64 formats are supplemented. In this supplementary experiment, the running time, F1 score, and model parameters were compared, and the optimal model was retrained with two datasets to obtain the recognition precision. The results are as Table 5.

## 6. Conclusions

In this paper, we focused on searching for HAR models with high computational efficiency and good performance by means of automatic network architecture design. We adopted a multi-objective NAS method based on NSGA-II to replace manual model design and accordingly proposed HARNAS. To solve the tradeoff problem between high efficiency and high performance, we first considered a bi-objective method in which the search targets were based on the weighted F1 score and the number of FLOPs. Later, because previous studies have found that the efficiency of a model is related not only to its complexity, but also to its memory use, the bi-objective search task was extended to a tri-objective task by adding an objective based on the MAC. Finally, using the proposed tri-objective search strategy based on NSGA-II, the Pareto optimal model with the optimal weighted F1 score could be found. At the same time, the proposed search strategy based on NSGA-II was able to find models with satisfactory transferability.

In addition, we can draw some other minor conclusions from our numbers of channels. As the number of channels increases, both the model complexity and MAC increase. Meanwhile, although the trend of the Error metric is not clear, it is evident that even if the number of shallow network channels is increased, the network performance may not be improved. These findings also show that the number of shallow channels is a very sensitive parameter that needs to be evaluated for different tasks before implementation.

Considering the mechanism of detection of abnormal traffic and that phones and embedded platforms are considered as targets, it is necessary to provide real-time identification of abnormal traffic so as to capture abnormal behavior more quickly. So, the challenge ahead will be finding a balance between a low footprint (energy consumption and chip footprint) and throughput and processing time [34]. 

## Figures and Tables

**Table 1:** The types of operations in the search space (Conv. for convolution).

Operation	Kernel Shape	Layers and Parameters	Values
Convolutional phase	N × 1	2D Conv. kernel size; 2D Conv. padding size	(11,1), (7,1), (5,1), (3,1), (1,1) (5,0), (3,0), (2,0), (1,0), (0,0)
	N × N	2D Conv. kernel size; 2D Conv. padding size	(7,7), (5,5), (3,3) (3,3), (2,2), (1,1)
Dilated Convolutional phase	N × N	2D Conv. kernel size; 2D Conv. padding size; 2D Conv. dilation rate;	(3,3), (5,5) (2,2), (4,4) (2,2)
Average Pooling Max Pooling phase	-	pooling kernel size; pooling padding size;	(3,1), (1,3), (3,3) (1,0), (0,1), (1,1)
skip connection	-	-	

**Table 2:** The FLOP counts corresponding to the major activation function $C_{a}$.

Name	Equation	FLOPs
ReLU	f(x)=max(0,x)	1
sigmoid	$f\left(x\right)=\frac{1}{1+e^{-x}}$	4
tanh	$f\left(x\right)=\frac{e^{x}-e^{-x}}{e^{x}+e^{-x}}$	5

**Table 3:** On the Opportunity dataset, the number of internal nodes in the sought model structure was set to 2, 3, 4, or 5. Bi-objective and tri-objective searches were conducted to explore the influence of the architecture depth on the model performance.

		**Nodes = 2**			**Nodes = 3**		
Bi-objective	**Concat**	**Error**	**FLOPs**	**MAC**	**Error**	**FLOPs**	**MAC**
	1	9.203	453.822	0.215	9.080	504.070	0.273
	2	9.371	693.491	0.240	9.292	1012.617	0.490
	3	-	-	-	9.394	1243.817	0.440
	4	-	-	-	-	-	-
Tri-objective	1	9.347	343.490	0.117	9.034	581.052	0.326
	2	9.356	629.657	0.175	9.220	960.090	0.452
	3	-	-	-	9.425	1197.46	0.395
	4	-	-	-	-	-	-
		**Nodes = 4**			**Nodes = 5**		
Bi-objective	**Concat**	**Error**	**FLOPs**	**MAC**	**Error**	**FLOPs**	**MAC**
	1	9.007	640.422	0.340	9.158	564.878	0.344
	2	9.036	1023.15	0.540	9.000	883.453	0.440
	3	9.177	1324.275	0.511	9.223	1148.177	0.462
	4	-	-	-	9.750	1472.120	0.407
Tri-objective	1	8.972	643.806	0.386	9.066	498.816	0.275
	2	9.281	954.581	0.458	9.135	847.844	0.390
	3	9.365	1201.516	0.432	9.383	1041.329	0.365
	4	10.074	1710.425	0.615	9.611	1250.388	0.340

**Table 4:** The parameter values set when calculating the MACs of various operations.

Operation Name	Parameters		
	Padding (*P*)	Stride (*S*)	Expansion Rate (*D*)
Normal convolution	1	1	1
Dilated convolution	1	1	2
-	common hyperparameter: $H_{in}$ = 32 $W_{in}$ = 32 $C_{in}$ = 12 $C_{out}$ = 24		

**Table 5:** Comparison of the data representation precision with FP32 and FP64.

	Milliseconds/Iterations/Batch	Memory (MB)	F1 Score (%)
FP32	36.58	30.66	92.16
FP64	37.37	61.32	91.039

## Data Availability

Not applicable.
